# Minimally Invasive Versus Open Radical Antegrade Modular Pancreaticosplenectomy (RAMPS): A Multicenter Cohort Study on Surgical Radicality and Postoperative Outcomes

**DOI:** 10.3390/cancers18040633

**Published:** 2026-02-15

**Authors:** Lukas Heinrich Poelsler, Ruben Bellotti, Florian Primavesi, Eva Maier, Ines Fischer, Helwig Wundsam, Patrick Kirchweger, Stefan Schneeberger, Stefan Stättner, Matthias Biebl, Manuel Maglione

**Affiliations:** 1 Department of Visceral, Transplant and Thoracic Surgery, Center of Operative Medicine, Medical University of Innsbruck, 6020 Innsbruck, Austria; lukas.poelsler@i-med.ac.at (L.H.P.); ruben.bellotti@i-med.ac.at (R.B.); florian.primavesi@i-med.ac.at (F.P.); eva.maier@i-med.ac.at (E.M.); stefan.schneeberger@i-med.ac.at (S.S.); 2 Department of General, Visceral, and Vascular Surgery, Salzkammergut Klinikum Voecklabruck, 4840 Vöcklabruck, Austria; stefan.staettner@kepleruniklinikum.at; 3 Department of General, Visceral, Thoracic, Vascular and Transplant Surgery, Ordensklinikum Linz, 4010 Linz, Austria; ines.fischer@ordensklinikum.at (I.F.); helwig.wundsam@ordensklinikum.at (H.W.); patrick.kirchweger@ordensklinikum.at (P.K.); matthias.biebl@ordensklinikum.at (M.B.); 4 Klinik für Allgemein und Viszeralchirurgie, Kepler Universitätsklinikum GmbH Linz, 4020 Linz, Austria

**Keywords:** RAMPS, minimally invasive surgery, lymph nodes, pancreatic surgery, oncological outcome, pancreatic adenocarcinoma

## Abstract

**Introduction**: Radical Antegrade Modular Pancreatosplenectomy (RAMPS) was developed to improve surgical radicality for left-sided pancreatic ductal adenocarcinoma (PDAC). Although widely accepted, the optimal surgical approach—open versus minimally invasive (MI)—is still being debated. **Methods**: We conducted a multicenter retrospective cohort study across three Austrian centers, including all patients undergoing RAMPS between 2016 and 2023 indicated for suspected (pre-)malignant pancreatic lesions. Patients were grouped based on the surgical approach (MI vs. open). The primary endpoints were resection margin status and lymph node yield following PDAC resection. Secondary outcomes included survival for PDAC patients and postoperative complications; non-PDAC resections were also taken into account. **Results**: A total of 57 patients were included, of whom 34 had PDAC. In PDAC patients, the rate of tumor-free margins and the median lymph node yield were equivalent between the MI and open approaches (R0 rate: MI 92.9% vs. open 85%, *p* = 0.484; median lymph node yield: MI 16 (IQR 10–23) vs. open 19 (IQR 15–25), *p* = 0.314). Two-year overall survival was also comparable (MI: 71.6% vs. open: 66.4%, *p* = 0.479). Postoperative outcomes at 90 days, like CR-POPF and major complications (Clavien–Dindo ≥ IIIa), did not differ between the two approaches. MI-RAMPS showed non-significant favorable trends in median length of stay (*p* = 0.093) and likelihood of receiving adjuvant chemotherapy (*p* = 0.075). **Conclusions**: In our experience, MI-RAMPS demonstrates oncologic equivalence and similar early postoperative outcomes to open RAMPS, with potential advantages such as shorter length of stay and likelihood of receiving adjuvant chemotherapy.

## Simple Summary

We present our experience in the procedure of minimally invasive (MI) and open Radical Antegrade Modular Pancreatosplenectomy (RAMPS). For the resection of pancreatic ductal adenocarcinoma (PDAC), both surgical approaches produce similar results regarding R0 tumor resection rates and lymph node yields. Postoperative outcomes at 90 days are similar, even when non-PDAC patients are included in the analysis. The MI approach may be favored due to a shorter median length of stay and a higher probability of receiving adjuvant chemotherapy, although these differences are not statistically significant.

## 1. Introduction

A major surgical challenge in the treatment of malignancies of the pancreatic body and tail has long been the absence of standardized techniques to ensure clear tangential margins in the posterior plane to reduce posterior R1 resections and enhance adequate local lymph node retrieval. In response to these limitations encountered in standard retrograde pancreatosplenectomy (SRPS), Strasberg et al. introduced the Radical Antegrade Modular Pancreatosplenectomy (RAMPS) procedure in 2003 [1,2].

The dissection plane either includes Gerota’s fascia alone (anterior RAMPS, aRAMPS) or extends posteriorly to it, including the left adrenal if the tumor infiltrates beyond the fascia (posterior RAMPS, pRAMPS) [3,4]. Implementing this technique, the Strasberg group observed higher R0 resection rates, as well as higher numbers of retrieved lymph nodes [5]. In the past decades, RAMPS procedures have been increasingly applied, confirming their improved radicality compared to SRPS [6,7,8], although—despite a number of ongoing studies—no randomized controlled trial has been published so far [9,10,11].

Since its introduction in 1994 [12], minimally invasive (MI) distal pancreatectomy has steadily increased in popularity. Two recent randomized controlled trials confirmed its applicability, showing reduced time to functional recovery [13] and, even more importantly, non-inferiority regarding radicality in patients with resectable left-sided pancreatic cancer [14]. By contrast, high-level evidence data on MI versus open RAMPS are missing, and the available data are primarily based on studies with small sample sizes. Systematic reviews and meta-analyses of retrospective studies agree on the feasibility of MI-RAMPS, with comparable short- and long-term outcomes between the open and the MI approach [15,16,17], even though the number of yielded lymph nodes tends to be lower in MI-operated patients [18,19].

In this study, we present our experience with open and MI RAMPS in a retrospective multicenter analysis involving three Austrian institutions.

## 2. Materials and Methods

We conducted a multicenter retrospective cohort study using an individually and prospectively maintained institutional database from 1 January 2016 to 31 December 2023. Participating centers included the Department of Visceral, Transplant, and Thoracic Surgery at the Medical University of Innsbruck, the Department of General, Visceral, Thoracic, Vascular, and Transplant Surgery at Ordensklinikum Linz, and the Department of General, Visceral, and Vascular Surgery at the Salzkammergutklinikum Vöcklabruck, all located in Austria.

The study was reported in accordance with the STROBE guidelines for observational research [20]. It was conducted in compliance with the Declaration of Helsinki and approved by the ethics committee of the Medical University of Innsbruck (registry number: 1157/2024), as well as the local ethics committee of the Medical Faculty of Johannes Kepler University Linz (registry number: 1332/2024). The requirement for informed patient consent was waived by both committees due to the retrospective nature of the study.

### 2.1. Data Collection and Cohort Characteristics

We retrospectively analyzed all patients who underwent a RAMPS indicated for (pre-)malignant lesions during the study period, regardless of the final histopathological diagnosis. Data collected comprised age, sex, date of diagnosis, comorbidities, preoperative blood test results, tumor characteristics, including pancreatic lesion size, regional lymph node metastases, and resection margin status, as well as details of the surgical procedure, systemic treatment regimens, date and location of disease recurrence, and date of death or last follow-up within 90 days. Resection margin status (R) was defined based on the distance from tumor to resection edge and whether the margin was directly involved, classified by tumor, lymph node metastases, perineural invasion, or lympho-vascular invasion. Resection status was classified as R0 when no residual tumor was present, R1 when microscopic tumor involvement was detected within 1 mm of the resection margin [21], and R2 when a macroscopic tumor, either loco-regional or metastatic, was observed [22].

Patients were assigned to one of two groups according to the surgical approach: MI or open surgery. Whether the patient underwent MI or open surgery was at the surgeon’s discretion. All MI procedures were performed by hepatopancreatico-biliary surgeons trained in MI surgery.

The primary objective of the study was to compare surgical radicality between the two approaches, defined by resection margin status and the number of lymph nodes recovered, in PDAC patients only. Secondary oncological endpoints were disease-free survival (DFS), local recurrence rates, and overall survival (OS) from the date of surgery. Additionally, secondary endpoints focused on postoperative outcomes at day 90, including postoperative complications as defined by the International Study Group on Pancreatic Surgery (ISGPS), specifically the occurrence of clinically relevant postoperative pancreatic fistula (CR-POPF) [23], post-pancreatectomy hemorrhage (PPH) [24], and post-pancreatectomy acute pancreatitis (PPAP) [25]. Also included were length of stay, major complications classified as Clavien–Dindo grade IIIa or higher [26], and mortality within 90 days following surgery. For these secondary endpoints, all patients undergoing RAMPS, not only those diagnosed with PDAC, were taken into account.

### 2.2. Statistical Analysis

Statistical analysis was performed using IBM SPSS Statistics, Version 27 (SPSS Inc., Chicago, IL, USA). Continuous variables were presented as median values with interquartile ranges (IQRs), while categorical variables were expressed as frequencies and percentages. Comparisons between categorical variables were assessed using Pearson’s chi-square test or Fisher’s exact test, depending on sample size. Overall and disease-free survival analyses were performed using the Kaplan–Meier estimator and log-rank test. Survival was calculated from the date of surgery. Student’s *t*-test was used for continuous variables. A two-sided *p*-value of ≤0.05 was considered statistically significant.

## 3. Results

A total of 57 patients underwent the RAMPS procedure in the study period and were included in this analysis. Among these patients, PDAC was the most common histopathological diagnosis (n = 34).

For PDAC patients (see Table 1), open RAMPS was performed in 20 patients (58.8%); 14 patients (41.2%) underwent MI RAMPS. While the open group included seven posterior RAMPS procedures, all MI-RAMPSs were anterior procedures.

Demographic data did not differ significantly. There was a non-significant tendency towards more female patients in the MI group and more patients with tobacco consumption in the open group. Arterial resections were required in three cases (5.3%). In the MI case, the proper hepatic artery was resected and reconstructed in an end-to-end fashion following conversion. The other two cases were in the open RAMPS groups, with one patient having intraoperative iatrogenic damage of the superior mesenteric artery which needed to be reconstructed in an end-to-end fashion and the other being in need for celiac axis resection. Resections of the porto-mesenteric axis occurred in two cases (10.5%) and only in the open group. Postoperative course did not differ between the groups. A non-significant tendency towards higher rates of post-pancreatectomy hemorrhage (PPH) and shorter lengths of stay was observed.

As presented in Table 2, there were no differences between the two groups regarding TNM staging and grading. Also, there were no statistically significant differences in R0 resection rates and in the number of retrieved lymph nodes (85.0% R0 and 15.0% R1 in open RAMPS vs. 92.9% R0 and 7.1% R1 MI RAMPS; 19 nodes (IQR 15–25) in open RAMPS vs. 16 nodes (IQR 10–23) in MI RAMPS; *p* = 0.484 and *p* = 0.314, respectively). Five patients (four in the open group and one in the MI group) underwent surgery after an unexpected finding of an oligometastatic M1 situation was encountered intraoperatively (local peritoneal carcinosis in two patients and a subcapsular small liver metastasis in three patients).

Local recurrence was observed in five patients (35.7%) in the open group and in one patient (14.3%) in the MI group (*p* = 0.283). While all patients in the MI group (n = 14, 100%) received adjuvant chemotherapy, in 16 out of initially 20 patients (80%) in the open group, adjuvant chemotherapy could be administered within 3 months following resection (*p* = 0.075, see Table 2).

**Figure 1:**
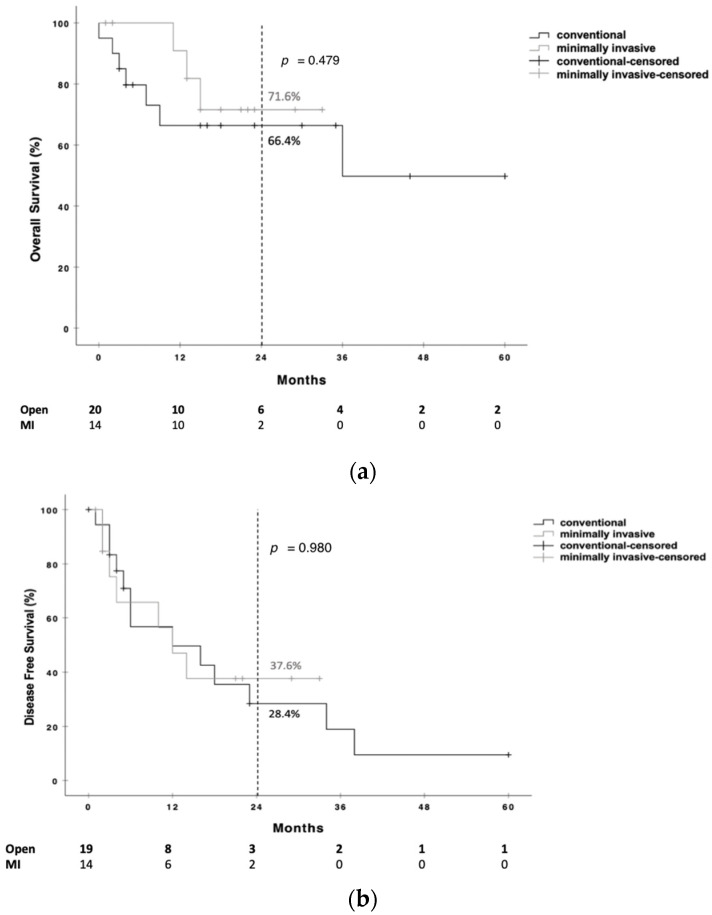
(**a**) Comparison of overall survival (OS) in PDAC patients undergoing open vs. MI RAMPS. (**b**) Comparison of disease-free survival (DFS) in PDAC patients undergoing open vs. MI RAMPS. Numbers at risk for each group are shown below the *x*-axis.

In terms of survival outcomes, following a median follow-up period of 666 (IQR 132–1049) days for open RAMPS and 490 (283–685) days for MI RAMPS (*p* = 0.846), the OS rate at two years was 66.4% and 71.6%, respectively, with no statistically significant difference (*p* = 0.479), as demonstrated in Figure 1a. The two-year DFS rate was 28.4% in the open RAMPS and 37.6% in the MI-RAMPS group, again without statistical significance (*p* = 0.980), as illustrated in Figure 1b.

For secondary endpoint analysis, all 57 patients undergoing RAMPS were included. Overall, 27 patients (47.4%) were female and 30 (52.6%) male. The median age was 63 years (IQR 30–84). Open RAMPS was performed in 33 patients (57.9%), 23 anterior and 10 posterior, while 24 patients (42.1%) underwent MI RAMPS.

A total of 40% of the patients had a final diagnosis different from PDAC. The most common non-PDAC diagnoses were neuroendocrine neoplasms (n = 12, 21.1%), intraductal papillary mucinous neoplasms (n = 4, 7.0%), and renal cell cancer (RCC) metastases (n = 2, 3.5%); one patient each (1.8%) had mixed neuroendocrine–non-neuroendocrine neoplasm (MiNEN), intraductal tubulopapillary neoplasm (ITPN), serous cystic neoplasm (SCN), solid pseudopapillary neoplasm (SPN), and acinar cell carcinoma.

Comparative analysis between open and MI RAMPS (see Table 3) revealed that patients in the MI group had significantly lower rates of tobacco use (0% vs. 21.2%, *p* = 0.016) and cardiovascular disease (8.3% vs. 30.3%, *p* = 0.045). Resections of the portomesenteric axis were exclusively performed in the open group (0% vs. 18.2%, *p* = 0.027). Pancreatic transection in the MI group was always performed with a stapling device, whereas techniques in the open group varied: in 6 cases (18.2%), the stump was oversewn, in 22 (66.7%) a stapler was used, and in 5 cases (15.2%), a combination of techniques was employed (*p* = 0.007).

Similarly to PDAC patients alone, the analyses of the entire patient cohort revealed comparable results for pancreatectomy-specific short-term outcomes, including the incidence of CR-POPF (30.3% in open RAMPS vs. 33.3% in MI RAMPS; *p* = 0.808), PPH (12.1% in open vs. 8.3% in MI; *p* = 0.122), and PPAP (6.1% in open vs. 0% in MI; *p* = 0.220). Also, the rates of relaparotomy within 90 days following surgery were equivalent between the two groups (15.2% in open RAMPS vs. 12.5% in MI RAMPS; *p* = 0.776), as was the incidence of major complications (36.4% in open vs. 41.7% in MI; *p* = 0.685) and wound infections (33.3% in open vs. 16.7% in MI; *p* = 0.158). The rates of hospital readmissions (24.2% in open vs. 25.0% in MI; *p* = 0.948) and 90-day mortality (9.1% in open vs. 0% in MI; *p* = 0.129) also showed no significant difference. In a similar vein, a non-significant tendency towards a shorter length of stay was observed.

## 4. Discussion

The present study supports the observation that MI RAMPS results in both short- and long-term outcomes comparable to those of the open approach. Specifically, the much-debated surgical radicality for PDAC did not differ between the two surgical accesses.

The open RAMPS approach yielded an 85% R0 resection rate, which, although marginally lower than that in the MI group, did not differ from it significantly. These results are consistent with the existing literature [5,16,19,27,28], and the tumor-free margin rates are comparable to those in a recent national French study, which defined a benchmark cut-off value of ≥75% for R0 resections in distal pancreatectomy [29]. In our study, we defined resection margins as R1 when microscopic tumor involvement was detected within 1 mm of the resection margin [21]. As the definition of resection margins for distal pancreatectomy remains controversial, with some studies suggesting that resection margins offer prognostic validity only in pancreatic head cancers and not in cancers of the body and tail [30,31], we decided not to subclassify resection margins further into R0, R1 (<1 mm), or R1 (direct involvement). In this study, this might not add additional information about surgical radicality. In the same vein, recent data from an Asian study and a European cancer registry described no prognostic difference between R0 wide and R1 < 1 mm resection margins in resected left-sided PDAC [32,33]. Over the 8-year period of case inclusion, pathological specimen examination varied and standardized processing was not available for all patients. Hence, it was not possible to report each margin (anterior, posterior and transection) separately.

Also, lymph node retrieval, the second key component of surgical radicality, did not differ significantly between the two groups. Both approaches yielded a median of more than 15 lymph nodes. In all cases in the open group, the minimum threshold of 15 lymph nodes recommended by the ISGPS [34] for adequate oncological staging was achieved. In the MI group, the range of yielded lymph nodes was wider, with a minimum number of 10. Despite the absence of statistical significance, this numerical discrepancy serves as a reflection of the ongoing discourse surrounding the radicality of the MI approach. While certain studies have documented higher lymph node yields in open surgery [16,18,19,35], others have indicated equivalence or enhanced outcomes with MI techniques following attainment of sufficient learning curves [15,28,36,37,38,39]. However, it is imperative to contextualize this debate within the broader framework of the significance of the number of yielded LN. In contrast to R0 resection, there is still a debate over whether nodal harvest should be seen as a staging rather than a curative procedure. Improved outcomes in patients with at least 20 lymph nodes examined have been reported [40,41]. However, missing information on systemic treatment and lymph node yield as a possible surrogate for surgical or hospital quality should be considered when interpreting these data. Other studies did not observe improved outcomes and see the advantage of the higher number of examined lymph nodes as referring to improved staging and less stage migration rather than to a “true” survival benefit [42,43]. With regard to extended lymphadenectomies, a recent study compared D1 versus D2 lymphadenectomy for left-sided PDAC. Despite the almost doubled number of examined lymph nodes, no differences could be seen in patient survival, nor in recurrence rate and recurrence location [44].

This may also be indicative of the mid- and long-term outcomes reported in the literature, which are largely comparable between the two approaches. High-level evidence supporting an oncological survival benefit following RAMPS compared to SRPS is still lacking, and the same is also true for open compared to MI RAMPS [9,10,11]. Our 2-year survival rates are similar between the two groups and align well with other reports supporting oncologic equivalence of the MI technique [16,18,19,28]. Even though not specifically addressing the RAMPS technique, the DIPLOMA study also showed a non-inferiority of the MI approach with similar OS and DFS, hence encouraging implementation of MI surgery for left-sided pancreatectomies if appropriate experience is available [14,45].

It is, however, important to note that the open approach was chosen more frequently for larger tumors and when involvement of major vascular structures was suspected. pT3 stages were more common in the open group, and posterior RAMPS and vascular resections were performed primarily in the open group. A similar discrepancy was also observed in a recent meta-analysis suggesting that surgeons prefer an open approach for larger tumors and for possible major vessel involvement [19]. Using a sophisticated propensity score matching, Ricci et al. showed worse short-term outcomes following MI surgery when patients were diagnosed with borderline resectable cases. For anatomically resectable tumors, MI RAMPS seems to be feasible and oncologically safe [15]. In light of the described feasibility of MI surgery for larger left-sided pancreatic tumors [46], they advocate for more studies addressing MI RAMPS in more advanced tumor stages.

Some statistically non-significant trends observed in postoperative outcomes, like shorter length of stay and lower rates of wound complications and pleural effusions, might favor the MI approach. Although these differences did not reach statistical significance, they align with existing studies reporting short-term benefits of MI techniques in pancreatic surgery [13,28,47,48]. Again, this needs to be interpreted with caution, as potentially more advanced tumors in the open group could have introduced a bias in these outcomes.

Major complications (Clavien–Dindo ≥ IIIa), as well as the rate of CR-POPF, occurred at similar rates between the groups, further supporting the equivalence of the MI approach. This is especially relevant in oncologic patients, for whom major complications may delay systemic therapy [49]. It is noteworthy that, despite the similar number of severe complications between the two groups, adjuvant chemotherapy was administered to all patients who underwent MI surgery. In contrast, when also including the three cases of postoperative mortality, 4 out of 20 patients undergoing open surgery did not receive adjuvant treatment. This may be due to the more advanced local stages in the open surgery group requiring more extensive surgery, or it may indicate the reduced time to functional recovery and improved quality of life following MI surgery in hepatobiliary–pancreatic resections [13,50]. The DIPLOMA trial found no difference in the administration of adjuvant treatment between the MI and open approaches [14].

Even though RAMPS has primarily been developed to optimize oncologic radicality for PDAC resections, in this study, 40% of patients had a final diagnosis different from PDAC. This probably reflects the relatively aggressive surgical approach adopted in cases with a strong suspicion of PDAC, combined with the frequent lack of diagnostically conclusive results from preoperative biopsy. In addition, RAMPS was also selected for large tumors with atypical imaging characteristics not clearly consistent with PDAC, or in order to ensure radical tumor resection despite, e.g., biopsy-proven pNEN.

Limitations should be considered when interpreting these findings. First, the relatively small sample size of the PDAC group, but also of the entire patient cohort, limits the statistical power and generalizability of the results. Second, the retrospective design is inherently associated with potential biases, including incomplete data collection, variability in perioperative management, and unmeasured confounding factors, including a potentially imbalanced disease stage between groups. Furthermore, laparoscopic and robotic-assisted surgery were included in the same group, vascular resections were predominantly performed in the open and not in the MI group, and information on the resectability status at presentation is not available. This disparity may have introduced selection bias and could partially account for the observed non-inferiority of the MI approach.

Surgical experience is a critical factor in complex pancreatic procedures. Across the three participating centers, MI RAMPS was performed by surgeons experienced in MI surgery within high-volume pancreatic surgery programs, with an annual case-load of approximately 40 to 100 pancreatic resections. At the smallest center, the pancreatic surgery program was led by two experienced surgeons previously affiliated with the Medical University of Innsbruck, resulting in a level of surgical expertise comparable to the two larger centers.

## 5. Conclusions

This multicenter retrospective analysis confirms that MI RAMPS offers oncologic outcomes non-inferior to the open approach in terms of resection margins, lymph node retrieval, and survival, with potential benefits for earlier physical recovery. It supports the safety and feasibility of MI-RAMPS in routine clinical practice, particularly if appropriate expertise in MI surgery within a high-volume center is available. Future prospective, multi-institutional, ideally randomized controlled studies with larger cohorts and stratification by tumor stage and surgical complexity are necessary to provide stronger evidence for surgical decision-making in left-sided pancreatic cancer.

## Figures and Tables

**Table 1:** Demographic, clinical, and perioperative characteristics of patients with PDAC stratified by open and MI RAMPS.

Value	Open RAMPS (n = 20)	MI RAMPS (n = 14)	*p*-Value
Female sex	8 (40.0)	10 (71.4)	0.092
Age (years) *	69 (58–75)	65 (57–74)	0.787
BMI (kg/m^2^)	25.3 (23.5–27.6)	25.0 (21.5–28.7)	0.491
Diabetes mellitus			0.782
Type I	3 (15.0)	1 (7.1)	
Type II	4 (20.0)	3 (21.4)	
Tobacco consumption	5 (25.0)	0 (0.0)	0.063
Alcohol abuse	0 (0.0)	0 (0.0)	n.a.
Cardiovascular disease	8 (40.0)	2 (14.3)	0.141
Neoadjuvant chemotherapy	6 (30.0)	3 (21.4)	0.704
ASA classification			0.162
1	2 (10.0)	1 (7.1)	
2	5 (25.0)	8 (57.1)	
3	13 (65.0)	5 (35.7)	
RAMPS operation technique			<0.001
Open anterior	13 (65.0)	0 (0.0)	
Robotic-assisted anterior	0 (0.0)	3 (21.4)	
Laparoscopic anterior	0 (0.0)	11 (78.6)	
Open posterior	7 (35.0)	0 (0.0)	
Operation time (min) *	263 (182–321)	292 (191–337)	0.798
Arterial resections ^#^	2 (10.0)	1 (7.1) ^§^	0.661
PV/SMV resections	2 (10.0)	0 (0.0)	0.501
Pancreas transection method			0.129
Stump oversew	3 (15.0)	0 (0.0)	
Stapler	15 (75.0)	14 (100)	
Stapler + stump oversew	2 (10.0)	0 (0.0)	
Use of sealants	4 (20.0)	3 (21.4)	0.622
Falciforme/omental flap	10 (50.0)	8 (57.1)	0.738
Perioperative somatostatine	1 (5.0)	0 (0.0)	0.588
Perioperative corticosteroids	0 (0.0)	0 (0.0)	n.a.
Postoperative hospital stay (days) *	15 (10–25)	12 (9–14)	0.104
Completion pancreatectomy	0 (0.0)	0 (0.0)	n.a.
POPF			0.850
0	9 (45.0)	6 (42.9)	
Biochemical leak	4 (20.0)	2 (14.3)	
B	6 (30.0)	4 (28.6)	
C	1 (5.0)	2 (14.3)	
PPAP	2 (10.0)	0 (0.0)	0.501
PPH			0.058
0	16 (80.0)	12 (100)	
A	4 (20.0)	0 (0.0)	
B	0 (0.0)	0 (0.0)	
C	0 (0.0)	2 (14.3)	
Postoperative blood transfusions	4 (20.0)	2 (14.3)	0.518
Wound complications	7 (35.0)	3 (21.4)	0.467
Postoperative paralysis	0 (0.0)	1 (7.1)	0.412
Pleura effusion	3 (15.0))	2 (14.3)	0.672
90-day relaparotomy	4 (20.0)	3 (21.4)	0.622
Complications according to Clavien–Dindo classification			0.214
0	7 (35.0)	1 (7.1)	
I	0 (0.0)	2 (14.3)	
II	5 (25.0)	4 (28.6)	
IIIa	3 (15.0)	2 (14.3)	
IIIb	4 (20.0)	5 (35.7)	
IVa	0 (0.0)	0 (0.0)	
IVb	0 (0.0)	0 (0.0)	
V	1 (5.0)	0 (0.0)	
Readmission	7 (35.0)	5 (35.7)	0.623
90-day mortality ^+^	3 (15.0)	0 (0.0)	0.251

* median (IQR). ^#^ open: proper hepatic artery, 1; superior mesenteric artery, 1. MI: proper hepatic artery, 1. ^§^ converted for arterial reconstruction. ^+^ 2, multi-organ failure by atypical pneumonia; 1, progressive disease. ASA: American Society of Anesthesiologists Physical Status Classification System; CR: clinically relevant; ERCP: endoscopic retrograde cholangio-pancreatography; PDAC: pancreatic ductal adenocarcinoma; PPAP: post-pancreatectomy acute pancreatitis; PPH: post-pancreatectomy hemorrhage; POPF: postoperative pancreatic fistula; PV: portal vein; SMV: superior mesenteric vein.

**Table 2:** Differences between open and MI RAMPS concerning oncologic parameters of PDAC patients.

Value	Open RAMPS (n = 20)	MI RAMPS (n = 14)	*p*-Value
T stage			0.301
pT1	2 (10.0)	1 (7.1)	
pT2	9 (45.0)	10 (71.4)	
pT3	9 (45.0)	3 (21.4)	
Resected lymph nodes *	19 (15–25)	16 (10–23)	0.314
N stage			0.576
pN0	9 (45.0)	7 (50.0)	
pN1	7 (35.0)	6 (42.9)	
pN2	4 (20.0)	1 (7.1)	
M stage			0.335
cM0	16 (80.0)	12 (92.3)	
cM1 ^#^	4 (20.0)	1 (7.7)	
Local infiltration			
Vascular (V1)	3 (15.0)	3 (21.4)	0.628
Lympho-vascular (L1)	8 (40.0)	5 (35.7)	0.800
Perineural (Pn1)	13 (65.0)	10 (71.4)	0.693
Resection status			0.484
R0	17 (85.0)	13 (92.9)	
R1	3 (15.0)	1 (7.1)	
Histological grading			0.842
G1	1 (5.0)	1 (7.1)	
G2	12 (60.0)	7 (50.0)	
G3	7 (35.0)	6 (42.9)	
Adjuvant chemotherapy (within 3 months following RAMPS)	16 (80.0)	14 (100.0)	0.075
Relapse	14 (70.0)	7 (50.0)	0.283
Local	5 (35.7)	1 (14.3)	
Systemic	9 (64.3)	6 (85.7)	
Follow-up (days) *	666 (132–1049)	490 (283–685)	0.421

* median (IQR). ^#^ M1: 2 patients with local peritoneal carcinosis, 3 with subcapsular small liver metastasis. PDAC: pancreatic ductal adenocarcinoma.

**Table 3:** Comparison of technical, operative, and perioperative characteristics between open and MI RAMPS.

Values	Open RAMPS (n = 33)	MI RAMPS (n = 24)	*p*
Female sex	10 (41.7)	14 (58.3)	0.157
Age (years) *	67 (30–81)	59 (30–84)	0.162
BMI (kg/m^2^) *	24.8 (19.7–48.7)	26.0 (18.1–37.7)	0.365
Diabetes mellitus			0.750
Type I	3 (9.1)	6 (18.2)	
Type II	1 (4.2)	4 (16.7)	
Tobacco consumption	7 (21.2)	0 (0.0)	0.016
Alcohol abuse	0 (0.0)	0 (0.0)	n.a.
Cardiovascular disease	10 (30.3)	2 (8.3)	0.045
Pulmonary disease	6 (18.2)	2 (8.3)	0.291
Neoadjuvant chemotherapy	8 (24.2)	3 (12.5)	0.267
Neoadjuvant radiotherapy	1 (3.0)	0 (0.0)	0.390
ASA classification			0.135
1	3 (9.1)	2 (8.3)	
2	15 (45.5)	17 (70.8)	
3	15 (45.5)	5 (20.8)	
Preoperative biopsy	7 (21.2)	7 (29.2)	0.491
Histological diagnosis			0.558
Pancreatic ductal adenocarcinoma	20 (60.1)	14 (58.3)	
Acinar cell carcinoma	1 (3.0)	0 (0.0)	
Intraductal papillary mucinous neoplasm	2 (6.1)	2 (8.3)	
Intraductal tubulopapillary neoplasm	1 (3.0)	0 (0.0)	
Renal cell carcinoma metastasis	2 (6.1)	0 (0.0)	
MiNEN	0 (0.0)	1 (4.2)	
Neuroendocrine neoplasms	6 (18.2)	6 (25.0)	
Serous cystic neoplasm	1 (3.0)	0 (0.0)	
Solid pseudopapillary neoplasm	0 (0.0)	1 (4.2)	
Operation time (min) *	258 (110–438)	268 (104–376)	0.594
Arterial resections ^#^	2 (6.1)	1 (4.2) ^§^	0.752
PV/SMV resection	6 (18.2)	0 (0.0)	0.027
Pancreas transection method			0.007
Stump oversew	6 (18.2)	0 (0.0)	
Stapler	22 (66.7)	24 (100)	
Stapler + stump overview	5 (15.2)	0 (0.0)	
Perioperative transfusion	4 (12.1)	2 (8.3)	0.645
Use of sealants	8 (24.2)	3 (12.5)	0.267
Falciforme/omental flap	18 (54.5)	14 (58.3)	0.776
Perioperative somatostatine	1 (3.0)	0 (0.0)	0.390
Perioperative corticosteroids	0 (0.0)	0 (0.0)	n.a.
Postoperative hospital stay (days) *	12 (7–47)	9.5 (5–28)	0.093
Completion pancreatectomy	0 (0.0)	0 (0.0)	n.a.
CR-POPF	10 (30.3)	8 (33.3)	0.808
PPAP	2 (6.1)	0 (0.0)	0.220
PPH	4 (12.1)	2 (8.3)	0.122
Postoperative blood transfusion	4 (12.1)	2 (8.3)	0.645
Wound complications	11 (33.3)	4 (16.7)	0.158
Postoperative paralysis	0 (0.0)	1 (4.2)	0.237
Pleural effusion	7 (21.2)	2 (8.3)	0.188
90-day relaparotomy	5 (15.2)	3 (12.5)	0.776
Clavien–Dindo > IIIa	12 (36.4)	10 (41.7)	0.685
Readmission	8 (24.2)	6 (25.0)	0.948
90-day mortality ^+^	3 (9.1)	0 (0.0)	0.129

* median (IQR). ^#^ open: proper hepatic artery, 1; superior mesenteric artery, 1; MI: proper hepatic artery, 1. ^§^ converted for arterial reconstruction. ^+^ 2, multi-organ failure by atypical pneumonia; 1, progressive disease. ASA: American Society of Anesthesiologists Physical Status Classification System; CR: clinically relevant; PPAP: post-pancreatectomy acute pancreatitis; PPH: post-pancreatectomy hemorrhage; POPF: postoperative pancreatic fistula; PV: portal vein; SMV: superior mesenteric vein. MI RAMPS included 19 laparoscopic and 5 robotic-assisted procedures. All MI procedures were anterior RAMPS.

## Data Availability

The datasets presented in this article are not readily available due to technical limitations. Requests to access the datasets should be directed to Manuel Maglione.

## Abbreviations

The following abbreviations are used in this manuscript:

aRAMPS	Anterior radical antegrade modular pancreaticosplenectomy
CR-POPF	Clinically relevant postoperative pancreatic fistula
DFS	Disease-free survival
ISGPS	International Study Group on Pancreatic Surgery
LOS	Length of stay
MI	Minimally invasive
O	Open (surgery)
OS	Overall survival
pRAMPS	Posterior radical antegrade modular pancreaticosplenectomy
PDAC	Pancreatic ductal adenocarcinoma
PPAP	Post-pancreatectomy acute pancreatitis
PPH	Post-pancreatectomy hemorrhage
RAMPS	Radical antegrade modular pancreaticosplenectomy
SRPS	Standard retrograde pancreatosplenectomy
